# Necrotizing fasciitis and fatal septic shock associated with *Streptococcus constellatus*

**DOI:** 10.4322/acr.2023.467

**Published:** 2024-01-08

**Authors:** Fareed Rajack, Shawn Medford, Tammey Naab

**Affiliations:** 1 Howard University Hospital, Department of Pathology and Laboratory Medicine, Washington, D.C., United States of America; 2 Howard University College of Medicine, Washington, D.C., United States of America

**Keywords:** Bacteremia, Debridement, Diabetic Ketoacidosis, Skin Ulcer, Streptococcus anginosus

## Abstract

*Streptococcus constellatus* is usually a benign, commensal bacteria but has increased incidence in blood cultures and abscesses. This pathogenic involvement is most prevalent in individuals with underlying medical conditions, such as solid tumors and type 2 diabetes mellitus, as well as in cases of community-acquired infections. We report a 43-year-old male with a right medial thigh ulcer and necrotic scrotal skin. The wound culture from surgical debridement grew *Streptococcus constellatus,* and histology was consistent with stage III necrotizing fasciitis. Regardless of etiology, the mortality rate of patients with necrotizing fasciitis is greatly decreased with early intervention and thorough surgical debridement.

## INTRODUCTION

*Streptococcus anginosus* group consists of three species: *S. anginosus*, *S. intermedius*, and *S. constellatus*, normal flora in the human oral cavity, gastrointestinal and urogenital tracts.^1^ Although rarely pathogenic, they are opportunistic pathogens, who tend to invade tissue and form abscesses, when impaired host defenses provide an opening. While SAG directly causes noninvasive infections, invasive infections can occur after entry into normally sterile areas of the body.^2^ The medical community is becoming aware of the increasing incidence of the group in blood cultures and abscesses. Virulence factors found in other streptococcal species are also present in SAG.^3^ Part of the challenge of identifying SAG infections when they occur lies in the wide variety of phenotypes and antigens in the group and their seemingly benign commensal presence.^4^

The abscesses commonly attributed to *S. constellatus* are one causality that may lead to the rare incidence of bacteremia, evolving into septic shock and a fatal outcome. The most common locations of the abscesses include the abdominal cavity, skin, orofacial region, urogenital tract, and the lower respiratory tract. These may spread by a hematogenous route to distant regions and form abscesses in distant locations, including the brain, liver, and heart, leading to endocarditis.^5^ The metastatic abscesses could lead to septic emboli, septic thrombophlebitis, or septic shock.^1^

## CASE REPORT

We report the case of a 43-year-old male presenting with a 5-day history of malaise and diarrhea. His relatives reported altered mental status. He had a history of a persistent right thigh wound of unknown duration. He had a history of schizoaffective disorder and bipolar disorder and was diagnosed with type-2 diabetes mellitus 20 years before, for which he took no medication. The patient presented with class III obesity with a BMI of 59.8.

Vital signs were unremarkable, with blood pressure 109/84, heart rate 86, respiratory rate 19, and temperature 37°C. The physical examination revealed a right medial thigh ulcer with necrotic scrotal skin and surrounding skin showing erythema and warmth to the touch. Pertinent abnormal admitting labs included blood glucose 401 mg/dL (Reference range [RR]: 70-99 mg/dL), Hb1Ac 12% (RR: 4-5.6%), beta-hydroxybutyrate 37.5 mg/dL (RR: <4.2 mg/dL), venous pH 7.29 (RR: 7.32 to 7.41), HCO_3_^-^ 17 mEq/L (RR: 23-26 mEq/L), anion gap 17 mEq/L (RR: 7-13 mEq/L), WBC 18.2 k/uL (RR: 4.5-11 k/uL), PTT 36.8 sec (RR: 25-35 sec), and INR 1.36 (RR: 0.8-1.1). Urine was negative for ketones.

**Figure 1:**
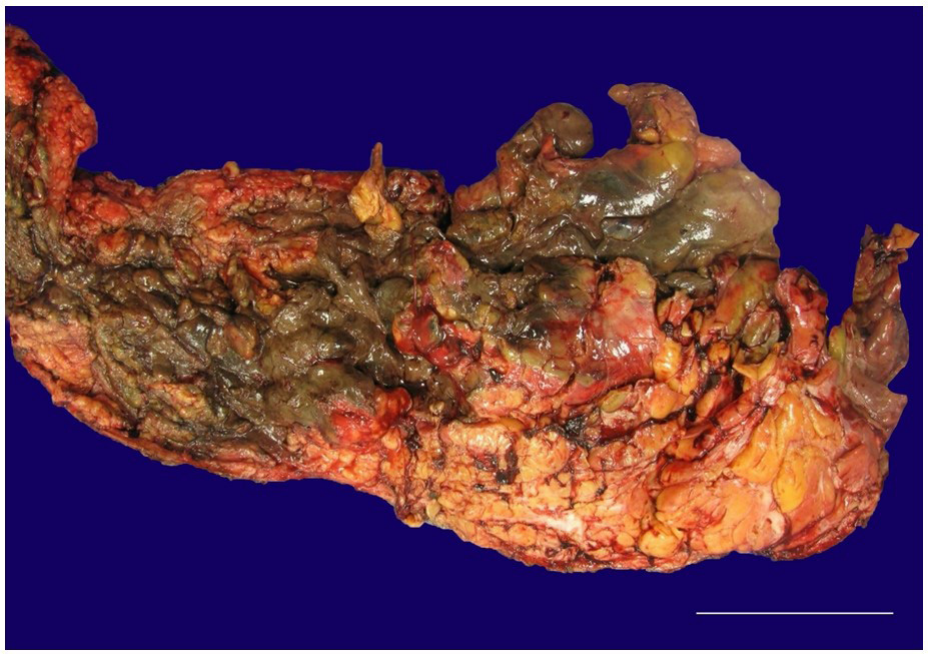
Gross pathologic examination of the debrided necrotic tissue measuring 44 × 17 × 4 cm (scale bar = 5 cm).

Empiric antibiotics were started in the emergency department. A general surgery consultant diagnosed necrotizing fasciitis. He underwent an urgent surgical procedure with right medial thigh and perineum excision and debridement. Blood loss was estimated to be 1 liter. Postoperatively, he received blood transfusions. He died less than one hour after returning to the surgical intensive care unit. The clinical cause of death was septic shock. We are reporting a case of fatal necrotizing fasciitis due to *Streptococcus constellatus,* which is confirmed by the gross and microscopic findings. Diabetic ketoacidosis was his significant risk factor. Two sets of blood cultures were negative. Deep wound culture grew *Streptococcus constellatus*. Debrided tissue measured 44 × 17 × 4 cm (Figure 1).

**Figure 2:**
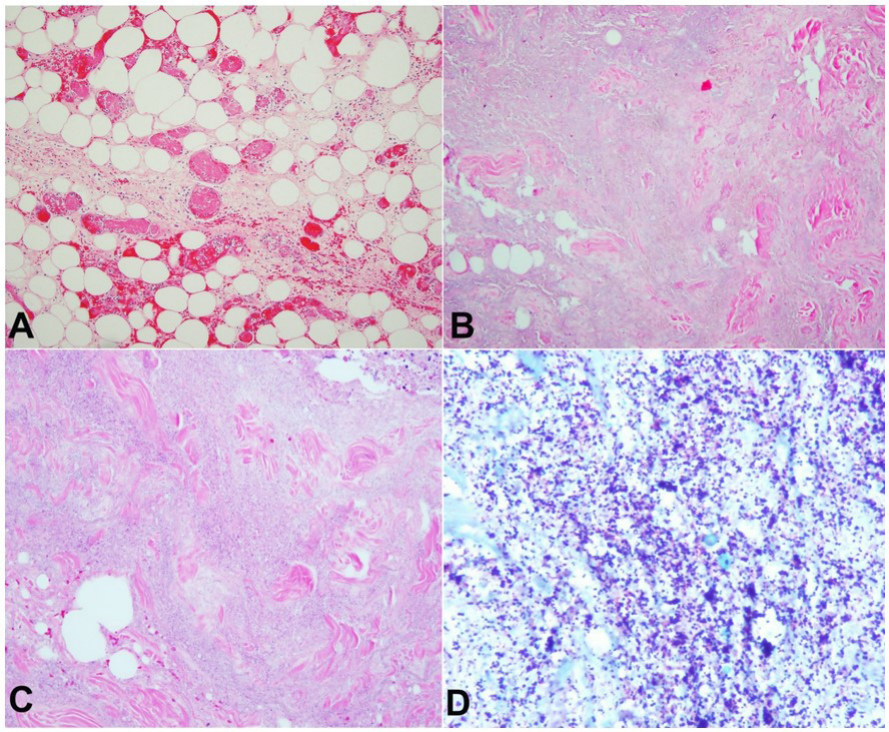
Photomicrograph of the surgical specimen. **A –** displays fat necrosis with fibrin thrombi in the blood vessels (H&E, 200X); **B –** displays pale gray necrotic tissue having a granular appearance without associated neutrophils consistent with stage III necrotizing fasciitis (H&E stain, 100X); **C –** displays pale gray necrotic tissue having a granular appearance without associated neutrophils consistent with stage III necrotizing fasciitis (H&E stain, 100X); **D –** shows sheets of gram-positive cocci (Gram stain, 100X).

Fibrin thrombi were present in viable subcutaneous adipose tissue (Figure 2A). Histology revealed abscesses next to zones of pale gray necrotic tissue having a granular appearance without associated neutrophils (Figures 2B and 2C). Gram stain of these pale gray zones revealed sheets of gram-positive cocci (Figure 2D), consistent with stage III necrotizing fasciitis (NF).

## DISCUSSION

While bacteremia is not a common finding for the group, it occurs more often with *S. anginosus* than with *S. constellatus* and usually involves patients with underlying conditions that predispose to opportunistic infections.^5^ In a case series of 51 patients with *S. anginosus* group bacteremia, 4 patients displayed signs of shock (7.8%), 2 patients had a fatal outcome (3.9%).^5,6^ An additional analysis of 19 patients with bacteremia involving the *Streptococcus anginosus* group found the mortality rate to be 26%.^5,7^

A 2020 retrospective analysis of 463 patients with *S. anginosus* group infections was conducted to identify the clinical characteristics of both the group and the individual species. Underlying medical conditions were identified in 210 of the 463 patients (45%), including solid tumors (30%) and type 2 diabetes mellitus (33%); community-acquired infections were present in 432 of the patients (93%). *S. constellatus* comprised 173 of the 463 specimens (37.4%). It was found that *S. constellatus* was more likely to infect the thorax than other areas and had a higher prevalence in the 35-54 age group than other age groups. Despite this, it is still inconclusive that any of the SAG species has a predilection for a specific area, and any age group may be infected with SAG, although there is a male predominance.^2^

Virulence is related to the production of hydrolytic enzymes (hyaluronidase, deoxyribonuclease, chondroitin sulfatase), the ability to bind fibronectin, and the presence of pyrogenic exotoxins and/or superantigens.^8^ The group’s well-known ability to form abscesses has been attributed to its polysaccharide capsule and synergism with anaerobic bacteria.^5,9^ One study demonstrated the ability of all encapsulated SAG isolates to avoid phagocytosis by polymorphonuclear neutrophils (PMNs) and to lead to subcutaneous abscesses in mice, whereas only 10% of unencapsulated isolates demonstrated the same capability. It also demonstrated that a higher concentration of capsular material in *S. constellatus* was proportional to phagocytotic inhibition.^10^ A recent study investigated the virulence of 41 SAG isolates by the activity of hemolysins, DNAses, and proteases. It found that the strains’ virulence factors allowed bacteria to evade immune system elimination and allow it to colonize the body.^11^

Whole-genome comparative analyses were conducted among 59 *Streptococcus* genomes and 7 SAG genomes, including 3 of *S. constellatus*. It identified many virulence factor homologs, such as LPxTG cell wall proteins, histidine kinases utilized in virulence gene regulation, adherence factors, invasion factors, and spreading factors. More than 10% of the entire SAG genomes were found to be comprised of bacteriophage and integrative conjugative mobile elements, and both single nucleotide polymorphisms (SNPs) and variable number tandem repeat (VNTR) changes were identified for *S. constellatus*. These findings serve as background for further investigation into the pathogenesis and virulence of SAG as well as both rapid diagnostics and therapeutics.^12^

SAG species are comprised of catalase-negative, non-motile facultative anaerobes. Isolates can be further identified as small colonies ≤0.5 mm in diameter with the group F antigen.^13^ Strains may be classified as Lancefield Group A, C, G, F, or have no Lancefield antigen.^4^ SC may be alpha or beta-hemolytic.^14^ However, SC is more commonly beta-hemolytic, unlike *S. intermedius*, which is usually alpha-hemolytic.^2^ Colonies often have a butterscotch or caramel-like odor.^13^ In our case, *S. constellatus* caused necrotizing fasciitis and fatal toxic Shock-Like Syndrome.

Necrotizing fasciitis can be classified into four different categories. Type I is polymicrobial, attributing for 70-80%, of cases and consists of mixed aerobes and anaerobes. Type II is monomicrobial, attributing of 10-20% of cases and comprised mostly of Group A *Streptococcus*, which *S. constellatus* is not a part of. Type III is marine-related gram-negative bacteria which are rare and are attributed to *Vibrio* species. Type IV is trauma-related which is rare and is caused by fungi.^14^ Only type II necrotizing fasciitis is associated with TSLS.

Type I represents the most common subtype, accounting for the cause of 55-75% of necrotizing soft tissue infections, most commonly affecting the trunk and perineal regions in immunocompromised patients such as those with peripheral vascular disease and diabetes.^15,16^ Type II infections often occur in young, healthy individuals and are seen much less frequently than type I infections. They are most commonly caused by *Streptococcus pyogenes*, commonly referred to as group A *Streptococcus*. This infection may also be in conjunction with *Staphylococcus aureus*.^15^ *S. pyogenes* employs a number of virulence factors such as M-1 and M-3 surface proteins, streptococcal pyogenic exotoxins (SPEs) A, B, and C, streptolysin O, and streptococcal superantigen (SSA). M-1 and M-3 facilitate tissue adherence and provide resistance from neutrophil phagocytosis. SPEs and SSA result in cytokine release and eventually lead to hypotension.^15^ S pyogenes often inhabits tissues that are not hypoxic and survive despite antibiotic therapy since they may exist intracellularly within macrophages.^15,17,18^

A collective review of 68 patients with necrotizing fasciitis in Turkey over 10 years identified *Escherichia coli*, *Enterococci*, and *Pseudomonas aeruginosa* as the most frequently isolated species.^19^ An additional review of 16 patients at Imperial College in London over 5 years found Group A *Streptococcus* to be the most common, with Group G *Streptococcus* and *Klebsiella pneumoniae* being other significant agents.^20^

The worldwide incidence of necrotizing soft tissue infections has a range from 1.69-15.5 cases per 100,000 person-years. There are about 1,000 cases of necrotizing soft tissue infections annually in the United States, equating to an incidence of 0.04 per 1,000 person-years. This has likely increased due to increased bacterial resistance and virulence, widespread antibiotic usage, and more accurate reporting.^15,16^ The mortality rate of necrotizing soft tissue infections is reported to be 25-35%, consistent with the rate over the past few decades.^15,21,22^

Necrosis usually occurs at a significant distance from the inciting wound. Due to the necrotic fascia, the skin and subcutaneous tissue can be separated from the fascia by applying light pressure. This can further progress to the deeper muscle layers, causing myonecrosis and myositis if treatment is not promptly initiated. Patients may often have limited sensation in the affected region because thrombosis of subcutaneous vessels leads to nerve fiber necrosis, resulting in anesthesia.^23^

The Laboratory Risk Indicator for Necrotizing Fasciitis (LRINEC) was developed to differentiate necrotizing fasciitis from other soft-tissue infections and was described first in a 2004 publication by Chin-Ho Wong et al.^24^ The hematologic and biochemical factors used as variables are C-reactive protein (CRP), total white cell count, hemoglobin (Hb), sodium (Na), creatinine (Cr), and glucose. These six parameters are given categorical values and are added to obtain a total LRINEC score. The authors concluded that patients with a LRINEC score ≥ 6 are to be assessed for necrotizing fasciitis.^24^ Sandner et al.^25^ evaluated this tool and found it to have a sensitivity of 0.94, a specificity of 0.94, a positive predictive value (PPV) of 0.29, and a negative predictive value of 0.99.^23,25^

Necrotizing fasciitis is reported at a higher frequency in immunocompromised patients with conditions such as cancers, vascular insufficiency, neutropenia, and type-2 diabetes mellitus.^23^ 76.4% of the 68 total patients were found to have comorbid conditions in the 2012 collective review in Turkey. The mortality rate of their patients was 13.2%, and they discussed early diagnosis as a critical factor to reduce mortality.^19^

MRI has been described as the preferred technique, largely due to its ability to portray soft tissue contrast, spatial resolution, and soft tissue fluid. Other imaging modalities that may be employed include ultrasound and computed tomography (CT). While CT often may not reveal early findings, it may be useful in identifying gas within tissues as well as asymmetrical fascial thickening, indicating necrosis. Radiography is not to be used as the soft-tissue gas observed may indicate gas produced by bacterial species unrelated to necrotizing fasciitis or gas released during surgical debridement.^23^ Ultrasonography has been demonstrated to show abnormalities in subcutaneous fat, investing fascia, and muscle, as well as indicate subcutaneous emphysema, fluid collections, and abscess formations.^23,26,27^

Diagnosis is made based on location (epicenter in subcutaneous fat and deep fascia) and histology (necrotic fascia with pale gray granular look, neutrophilic infiltration (PMN) in deep dermis and fascia), thrombosed viable microcirculation, and the infecting microbe being identified in necrotic tissue.^28^

Prognostic necrotizing fasciitis stages are based on histologic and Gram stain (GS) findings: stage I has moderate-to-severe neutrophilic infiltration and the absence of bacteria in infected tissue; stage II has either moderate-to-severe neutrophilic infiltration and the presence of bacteria in infected tissue or few/no neutrophilic infiltration and the absence of bacteria in infected tissue; and stage III has few/no neutrophilic infiltration and the presence of bacteria in infected tissue (Table 1).^29,30^

**Table 1:** Prognostic staging and clinical staging of necrotizing fasciitis. Prognostic staging is based on histology and Gram stain.^29^ Clinical staging is based on cutaneous manifestations^30^

	**Neutrophilic infiltration**	**Bacteria in infected tissue?**	**Cutaneous manifestations**
**Stage 1**	Moderate-to-severe	No	Tenderness to palpation, erythema, swelling, and warm skin
**Stage 2**	Moderate-to-severe	Yes	Blisters or bullae (serous fluid)
	Few/none	No	
**Stage 3**	Few/none	Yes	Crepitus, skin anesthesia, and skin necrosis with apparent discoloration

These stages were developed in a retrospective review of 82 cases of necrotizing fasciitis in which histopathological findings available for 63 cases were used to correlate histopathological findings with clinical outcomes. Mortality rates were found to be 7.1% for stage I, 14.2% for stage II, and 47% for stage III.^29^

A retrospective study of 22 patients with NF was conducted to create a clinical staging system based on cutaneous manifestations. Stage 1 (early) features were categorized as tenderness to palpation, erythema, swelling, and warmth. Stage 2 (intermediate) included the formation of blisters or bullae. Stage 3 (late) cutaneous features were crepitus, skin anesthesia, and skin necrosis with apparent discoloration. The authors concluded that such a staging system could help with early identification.^30^

Early intervention and the thoroughness of the initial surgical debridement are noted by many to be the most important factors to decrease mortality.^15^ In a study of 65 patients with necrotizing soft tissue infections secondary to complications from postoperative wounds, it was found that the average time elapsed from admission to operation was 90 hours for nonsurvivors compared to 25 hours for survivors.^23,31^ A retrospective review of 89 patients admitted for necrotizing fasciitis showed that those whose surgery was delayed over 24 hours from admission had a relative risk of mortality of 9.4.^15,32^

A study of 18 rheumatic disease patients with necrotizing fasciitis found that the relative risk of mortality was 7.5 times greater for patients with insufficient initial surgical debridement than those with sufficient initial debridement.^15,33^ A retrospective review of 68 patients found a mortality rate of 38% for patients in Group A, which either had delayed or inadequate initial treatment, versus a mortality rate of 4.2% in Group B, which had aggressive initial debridement.^15,34^

Our patient with a BMI of close to 60 presented with a body habitus that risks diminishing the quality of radiographic mage quality, requiring modifying techniques for image procurement. For example, large amounts of subcutaneous adipose tissue leads to attenuation of the ultrasound signal, requiring use of the lowest frequency probe. For X-rays, the greater distans that the x-ray beams need to travel may require incrased film speed and adjusted image settings. CT and MRI imaging may be limited by aperture diameter and table thickness. Lower perfusion in subcutaneous adipose tissue also increases risks of surgical site infections due to ischemia, necrosis, and impairments in leukocytes’ oxidase system. Furthermore, surgeons have reported increased procedural difficulty due to large body habitus of patients.^35-37^

## CONCLUSION

Concomitant necrotizing fasciitis and TSLS have a 60% mortality rate. Patients with obesity and uncontrolled DM have an even increased risk for mortality. Gram stain and culture of debrided tissue are essential. Rare virulent pathogens, like *Streptococcus constellatus*, can be causative in association with anaerobes. Early management and surgical debridement are paramount to improving patients’ prognosis with necrotizing fasciitis.
